# Evidence of learning in workplace-based assessments in a Family Medicine Training Programme

**DOI:** 10.4102/safp.v66i1.5850

**Published:** 2024-04-26

**Authors:** Neetha J. Erumeda, Ann Z. George, Louis S. Jenkins

**Affiliations:** 1 Department of Family Medicine and Primary Care, Faculty of Health Sciences, University of the Witwatersrand, Johannesburg, South Africa; 2 Gauteng Department of Health, Ekurhuleni District Health Services, Germiston, South Africa; 3 Centre of Health Science Education, Faculty of Health Sciences, University of the Witwatersrand, Johannesburg, South Africa; 4 Division of Family Medicine and Primary Care, Faculty of Medicine and Health Science, Stellenbosch University, Cape Town, South Africa; 5 Western Cape Department of Health, George Hospital, George, South Africa; 6 Primary Health Care Directorate, Department of Family, Community and Emergency Care, Faculty of Health Sciences, University of Cape Town, Cape Town, South Africa

**Keywords:** assessment for learning, assessment of learning, formative assessments, workplace-based assessments, work-based learning, family physician, family medicine, learning portfolio

## Abstract

**Background:**

Learning portfolios (LPs) provide evidence of workplace-based assessments (WPBAs) in clinical settings. The educational impact of LPs has been explored in high-income countries, but the use of portfolios and the types of assessments used for and of learning have not been adequately researched in sub-Saharan Africa. This study investigated the evidence of learning in registrars’ LPs and the influence of the training district and year of training on assessments.

**Methods:**

A cross-sectional study evaluated 18 Family Medicine registrars’ portfolios from study years 1–3 across five decentralised training sites affiliated with the University of the Witwatersrand. Descriptive statistics were calculated for the portfolio and quarterly assessment (QA) scores and self-reported clinical skills competence levels. The competence levels obtained from the portfolios and university records served as proxy measures for registrars’ knowledge and skills.

**Results:**

The total LP median scores ranged from 59.9 to 81.0, and QAs median scores from 61.4 to 67.3 across training years. The total LP median scores ranged from 62.1 to 83.5 and 62.0 to 67.5, respectively in QAs across training districts. Registrars’ competence levels across skill sets did not meet the required standards. Higher skills competence levels were reported in the women’s health, child health, emergency care, clinical administration and teaching and learning domains.

**Conclusion:**

The training district and training year influence workplace-based assessment (WPBA) effectiveness. Ongoing faculty development and registrar support are essential for WPBA.

**Contribution:**

This study contributes to the ongoing discussion of how to utilise WPBA in resource-constrained sub-Saharan settings.

## Background

The World Health Organization (WHO) advocates that medical training should develop socially accountable health professionals with the required skills.^1,2^ Socially accountable medical training requires education and service activities that address the priority health concerns of the communities being served.^2^ Trainees’ ‘social responsiveness’, an essential element of social accountability,^3^ refers to their ability to integrate the complex skills required to attend to patient needs and improve patient outcomes in the community.

The recent shift to work-based learning (WBL) and workplace-based assessment (WPBA) aligns with the call for greater social accountability in medical training. Training in authentic clinical contexts promotes professionalism, communication, teamwork and interprofessional collaboration and enhances self-directed learning, thus preparing trainees to be responsive to communities’ needs.^4,5^ Despite the benefits of WBL, medical schools may not be equipped to assess the complex integration of knowledge, skills and attitudes in authentic settings and have been criticised for not linking the phases of trainee learning with patient outcomes.^5^ The educational impact of WBL has been explored in high-income countries, but the use of WPBAs and the types of assessments used for and of learning have not been adequately researched in sub-Saharan Africa. This article describes the evidence of learning in WPBAs in a Family Medicine training programme in South Africa (SA).

Work-based learning shifts the focus from theoretical learning to learning in and from practice.^6^ Work-based learning also broadens the scope of educational assessment from assessment *of learning* (AoL), summative components with pass/fail decisions,^5^ to assessment *for learning* (AfL).^7^ Assessments *for learning* form part of day-to-day clinical practice: seeking, reflecting and responding to information from observation and dialogue to promote learning.^8^ Assessment *for learning* provides meaningful narrative feedback to enhance the learning experience.^7,9^ Assessments *of learning* are high-stake assessments focusing on achieving learning outcomes at the end of a programme^7,10^ for accountability, ranking and certification of trainee’s achievement of competence.^11^

The distinction between AfL and AoL is complex, making it difficult for trainers to discern how to use the different assessment types effectively.^11^ Workplace-based assessments captured in learning portfolios (LPs) provide adequate evidence of trainees’ competence and are collectively used for the summative AoL.^7^ Given that WBL and WPBA are recent developments, it may be more difficult for trainers to use AfL and AoL to the trainees’ best benefit.

## Learning portfolios for workplace-based assessment

In programmatic assessment, defined as ‘a consciously designed assessment system in which the longitudinal development is visible to the learner as usable feedback which provides rich data for informed, holistic decision on making learner progression’, individual data points from low-stake AfL accumulate into multiple data points for high-stake AoL.^4^ Learning portfolios, introduced in the United Kingdom in 1996 for postgraduate general practice vocational training,^12,13^ are now widely used as a longitudinal tool in programmatic assessments.

Learning portfolios offer several benefits for trainees’ longitudinal development. Portfolios may include evidence of personal and professional WBL,^14^ assessments of soft skills, like professionalism, communication, teamwork and ‘hard’ clinical procedural skills.^4,15^ Soft skills are better learned and assessed when opportunities are provided to address the complexities of clinical practice in the workplace.^4,14,15^ Learning portfolios provide a systematic overview of the tasks, expected levels of competency for the required tasks, perceived levels of competence (ability to perform the tasks) and areas needing improvement.^15^ Competency cannot be measured, but competence, an attribute of the person performing the task, can be measured.^16^ Learning plans, an essential component of LPs, encourage trainees to track their educational progress and stimulate reflection and feedback as critical skills in self-directed learning.^13,17^ Supervisors are expected to assist trainees in developing learning goals and support them in achieving their learning plans.^18,19^ The most critical factor influencing LP use in high-income countries was supervision that stimulates trainees’ reflection and promotes deep learning.^20,21^ The organisation of portfolios also affects their efficacy.^21^ Trainees require clear guidelines on the portfolio structure, format and content and how to avoid excessive paperwork.^21^

Workplace-based assessments are best used as part of a programmatic assessment approach; no single assessment method embodies all the required educational characteristics, such as reliability and validity, educational impact and acceptability.^22^ However, different WPBA tools have varying educational impacts across clinical contexts.^23,24,25^ In high-income countries, the effectiveness of WPBA tools relied on prior training on using the tools and the contexts where assessments were conducted.^22,24^ One factor affecting WPBA is the lack of consensus on the number of assessments used as AfL.^24^ Other factors arise from the trainee-tool interactions. For example, trainee-assessor relationships could promote lenient marking.^24^ Despite the challenges associated with WPBAs, the trainees and trainers agree that WPBAs provide valuable narrative feedback and augment trainee self-reflective skills, thus improving learning opportunities.^26^

Potential barriers to effective LP use include the paperwork required, finding senior staff to supervise procedures, ineffective supervision, the lack of faculty engagement^21^ and ticking boxes to indicate whether skills were performed.^17,27,28^ Ticking boxes does not provide feedback on the quality of performance.^20^ Other barriers that have been identified were the need for multiple assessors, inadequate supervisor and trainee training on WPBA tools, low trainee confidence and whether WPBAs are conducted *for* learning or *of* learning.^13,21,24^ In one study, trainees were concerned that delayed feedback after WPBAs would impact their results – supervisors could not always remember individual trainees.^28^

While WBL theories and models have been developed, variations in how trainees and supervisors utilise WPBA tools across settings need further research.^13^ Workplace-based assessment tools have been extensively investigated in high-income settings like the Netherlands and the United Kingdom, but there is a paucity of studies exploring their effect on trainee performance in low-middle-income contexts.^29^ There is a need for further characterisation of LP use, purpose, structure, evidence for mentoring and assessments across different contexts.^21^ Some authors have suggested a need to explore the influence of context on LP use to understand why implementation failed in some contexts and to identify and interrogate improvements.^21^

## The SA learning portfolio requirements

Learning portfolios were introduced SA as a WPBA tool in postgraduate family medicine (FM) training in 2012.^30^ The Colleges of Medicine of South Africa (CMSA), the examining board for the national exit examinations for registrars (SA postgraduate medical trainees), specifies five-unit standards and 83 programmatic learning outcomes to be achieved at the end of a 4-year specialist training programme.^30,31,32^ The national LP was designed to capture the evidence for 50 of the 83 learning outcomes.^31,33^ The LP records evidence of registrars’ personal and professional development through reflection and from WPBA tools.^30^ Detailed learning plans should specify registrars’ learning needs, plans to address their needs and the goals they have achieved.^34^ Registrars have to obtain a subminimum of 60% in the LP for each of 3 years to progress to the following year and qualify for the national exit examinations.^30^

The guidelines for the national LP recommend establishing effective supervisor-registrar relationships with regular engagements, including monthly meetings, at least 6 h of dedicated weekly training and supervision and adequate mentoring.^30^ Learning portfolios can be handwritten or electronic, depending on university requirements, but electronic portfolios are associated with better completion rates, improved quality of scoring entries, increased frequency of use and sustained supervisor-registrar engagement.^35^ Previous SA studies found that registrars’ reflective skills, knowledge of the requirements, good supervisor feedback and regular engagement with supervisors positively influenced LP use.^34^

The skills list for registrars was revised and validated in a 2018 national Delphi study.^36^ This list of 205 core and 39 elective skills is part of the national LP requirements.^30^

There are four self-reported competence levels for skills:

- A – Only possesses theoretical knowledge about the skill.
- B – Possesses theoretical knowledge and observed that skill performed.
- C – Performed the skill under supervision.
- D – Can be fully entrusted to perform that specific skill independently.

There are few studies on WPBA and using LPs in postgraduate medical training in low-middle countries, especially in public district health systems where WBL occurs across various primary health care (PHC) facilities and hospitals. This study adds to the discourse by quantifying evidence of learning in LPs, evaluating the AfL and AoL, and investigating the influence of context, such as the training district or the year of training, on the nature of assessments.

## Postgraduate family medicine programme at the University of the Witwatersrand

Family medicine was recognised nationally as a new speciality in 2007. The University of the Witwatersrand (Wits University) introduced a full-time postgraduate decentralised FM training programme in 2008. The training programme is structured across 4 years of training. Registrars undergo 2- to 3-week theoretical training blocks at the university annually, depending on the training year. The university also provides an online component comprising FM-related clinical and non-clinical topics, research and journal clubs. Fourth-year registrars focus on the research component and undergo elective rotations to gain further knowledge in a clinical discipline or an area, depending on the registrar’s interest gained during training years.

The FM registrar training programme is based in PHC clinics, community health centres (CHC) or district hospitals across the five study districts. Community health centres are 24-h nurse-led facilities supported by general practitioners and family physicians (FPs). District hospitals are 50–300 bedded hospitals serving a defined population and providing 24-h comprehensive care services and run by general practitioners with a few general specialists, including FPs.^37^ Registrars rotate every 2 to 3 months in the district or regional-hospital departments for skills training. Skills rotations occur with registrars rotating through various departments, including internal medicine, obstetrics and gynaecology and paediatrics. The timing, location and duration of clinical rotations vary between districts. Decentralised clinical training also includes weekly training sessions at district regional training centres comprising academic discussions (clinical and non-clinical topics), clinical case discussions (one-on-one or group) and WPBAs using the mini-clinical evaluation exercise (mini-CEX) or direct observation of procedural skill tool. The structure of these sessions varied slightly across districts. Another training component is the monthly or bimonthly one-on-one engagements, captured in the LP, between registrars and supervisors to discuss registrars’ learning needs, review their learning plans and progress and identify gaps to be addressed. In some districts, registrars also have observed consultations or procedures with FP supervision at CHCs or PHCs.

The University of the Witwatersrand FM registrar training uses the programmatic assessment model,^38^ with multiple assessments during the training year. Assessments *for learning* include WPBAs across the CMSA-prescribed curriculum captured in the LP. Other LP sections are learning plans, educational meetings, direct observations, written assignments, logbooks, certificates for attending courses, meetings and end-of-year assessments.^30^ Assessment *for learning* is used to decide annual registrar progression: the quarterly assessments (QA) and the LP overall score are considered. The QA are observed consultations or procedures evaluated using WPBA tools by a supervisor from another district. The QA scores were taken as AfL separately from the overall LP scores contributing to the year mark. The involvement of multiple supervisors improves the validity of WPBA.^24^ Assessment *of learning* includes university examinations conducted 18 months into the training programme and the national exit examination after at least three training years as the final high-stake assessment towards specialist qualification.

In the logbook section of the Wits University FM LP, registrars must achieve competencies clustered into core and elective skills within 14 clinical domains aligned to CMSA requirements. These clinical domains are adult health, ear, nose and throat (ENT), eyes and skin, women’s health, emergency care, child health, consultation skills, clinical governance, orthopaedics, anaesthetics, forensics, community-oriented primary care (COPC), clinical administration, teaching and learning and palliative care. Registrars must achieve competence at level D for all core skills and level C or D for elective skills. Their immediate supervisors evaluate registrars’ bi-annual self-assessments of skills in their logbooks before submitting the LPs to the university for final evaluation by the portfolio committee.

## Methods

### Study design

This study forms part of a broader, mixed-methods case study evaluating the postgraduate FM training programme at University of the Witwatersrand using a logic model. The quantitative component of the broader study reported in this article evaluated evidence of learning in WPBAs as short-term outcomes of the logic model – the short-term outcomes were proxy measures of registrars’ knowledge and skills.

### Study setting

The study was conducted across the five training districts affiliated with University of the Witwatersrand: Ekurhuleni, Johannesburg Metro, Sedibeng and West Rand in the Gauteng province and Dr Kenneth Kaunda in the North West province of South Africa. Gauteng province is more densely populated (total population above 15 million) than the North West province (just over 4 million).^39^ However, more than 75% of the population in both provinces is uninsured and utilises government health services at PHC clinics, CHCs and district hospitals.^39^ There is a gross disparity in the distribution of doctors across the provinces. While Gauteng province has over 4000 medical practitioners and 1500 specialists, North West has an acute doctor shortage with just above 1000 medical practitioners and 100 specialists covering the province.^39^

The health facilities that serve the population vary widely across the training districts.^39,40^ While the Johannesburg Metro district has 108 PHC clinics, 11 CHCs and two district hospitals, Ekurhuleni has 84 PHC clinics, nine CHCs and one district hospital. Sedibeng has 8 CHCs and 30 PHC clinics, while West Rand district has 45 PHC clinics and 3 CHCs.^41^ The less-populated Dr Kenneth Kaunda district in the North West province has only 30 PHC clinics and 10 CHCs, spread over 14 671 km.^41^ The number of regional and tertiary hospitals ranges from five hospitals in the Johannesburg Metro district to no tertiary facility in the Sedibeng, West Rand and Dr Kenneth Kaunda districts.^41^ Most of the uninsured population in these communities relies solely on health services provided by PHC and CHC facilities as the first point of health care, emphasising the need for high-quality patient care to be provided at these facilities.

### Study population and sampling

The study population included all 18 registrars’ LPs across 3 years of the 2020 training programme. All 18 registrars who submitted LPs at the end of the training year 2020 (*N* = 18) consented to their LPs being evaluated. The registrars’ total QA and LP sectional scores, including their skills competence levels and final LP scores, were collected as the AfL. The AoL component for the first- and second-year registrars was the outcome of the 18-month university examinations and the national exit college examination results for third-year registrars.

### Data collection

The ‘evidence for learning tool’ was developed to record registrars’ scores and skills competence levels. Quarterly assessment scores and university or CMSA examinations’ pass or fail outcomes (in the same or the following year) were extracted from university records. Registrars in the second year wrote the University of the Witwatersrand exams in 2020; the first years wrote in 2021 after completing their 18 months of training. The third-year registrars, who had submitted their LPs in 2020, sat the CMSA exams in 2021, in their fourth year of study. This study used supervisors’ total scores allocated for QA, LP and various LP sections and the final pass/fail in the university or CMSA examinations as proxy measures of registrars’ knowledge.

The nationally validated skills set^36^ was used to develop the skills section of the data collection tool. Registrars’ self-assessment scores for each skill in the LP logbook were taken as proxy measures for skill competencies. After the final submission, the registrar’s immediate supervisor and the university’s portfolio committee verified all the LP scores.

### Data analysis

The data were entered into an Microsoft Excel spreadsheet and imported into Stata 14.2 software. Median and interquartile ranges were calculated for the total LP, LP sectional and QA scores for each training district and year. Frequencies of self-reported competence levels for each skill (category scores A–D) were determined. Registrars’ total skill-set scores for each domain were calculated using the highest competency level ‘D’; competence levels A, B and C were not considered. For example, if a registrar assessed themselves at level D in 30 of the 38 ‘adult health skills’, their total skills set score for that domain would be 30/38. The median and interquartile ranges for each domain’s total skill set score were calculated for each training year and district.

### Ethical considerations

Ethical approval was obtained from the Human Research Ethics Committee (HREC Medical) of the University of the Witwatersrand (Certificate number M191140). Permission was obtained from the University Registrar and the Head of the Department of Family Medicine to conduct the research and access the university records and LPs. Informed consent was obtained from registrars to access the LPs. The research was carried out following the Helsinki Declaration.

## Results

Six LPs from each year of registrar training were assessed (*N* = 18). The total LP median scores and interquartile ranges were 76.3 (62.1–81.2), and the median and interquartile ranges for the QA scores were 64.9 (60.7–69.5).

The median and interquartile ranges calculated for the total LP scores and various LP sectional and QA scores across the training years (Y1–Y3) are shown in Table 1. The total LP median scores were higher in Year 3 compared to Year one (Y1) and Year two (Y2) (Table 1). The Year two registrar’s QA median scores were higher than Years one (Y1) and three (Y3) (Table 1). Supervisors awarded full marks (10/10) in some LP sections in Y1 and Y2 but not in Y3 (Table 1).

**TABLE 1:** Assessment for learning scores in learning portfolios and quarterly assessments across training years.

Type of assessments	Total scores	Assessment scores					
		Year 1 (Y1) (*n* = 6)		Year 2 (Y2) (*n* = 6)		Year 3 (Y3) (*n* = 6)	
		Median	IQR	Median	IQR	Median	IQR
**Learning portfolio sections**							
Learning plan (a)	10	7.8	7.0–8.3	9.2	8.0–10.0	8.2	7.5–8.7
Supervisor report on clinical allocations (b)	10	7.9	7.5–8.3	8.1	7.6–8.3	8.1	7.7–8.7
Educational meetings (c)	20	18.7	15.8–20.0	17.5	10.0–20.0	17.7	17.2–20.0
Observations of the registrar (d)	10	10.0	8.0–10.0	10.0	10.0–10.0	8.4	7.7–8.7
Written assignments (e)	10	6.4	6.0–6.6	7.0	6.8–7.2	6.5	5.8–6.7
Logbook (f)	30	0.0	0–20.0	20.8	14.0–30.0	23.7	21.5–27.0
Global rating (g)	10	7.0	6.0–8.0	6.0	6.0–6.0	7.0	6.0–7.0
Learning portfolio total scores (Total of rows a–g)	100	59.9	53.9–81.1	73.0	64–87	81.0	75.6–81.2
Quarterly assessment	100	61.4	55.7–67.1	67.3	62.6–73.2	64.8	62–67.5

IQR, interquartile range.

Table 2 shows the median and interquartile ranges calculated for the total LP scores and various LP sectional and QA scores across the five districts (D1–D5). The total LP median scores varied considerably across D1–D5 (Table 2), but not the QA median scores. Again, some LP sections (learning plans, educational meetings and observations) had median scores of 10/10 in some districts (Table 2).

**TABLE 2:** Assessment for learning scores in learning portfolios and quarterly assessments across training districts (*N* = 18).

Type of assessments	Total scores	Assessment scores									
		District 1 (*n* = 5)		District 2 (*n* = 3)		District 3 (*n* = 3)		District 4 (*n* = 4)		District 5 (*n* = 3)	
		Median	IQR	Median	IQR	Median	IQR	Median	IQR	Median	IQR
**Learning portfolio sections**											
Learning plan (a)	10	8.0	7.5–8.0	7.0	7.0–10.0	10.0	10.0–10.0	8.5	8.2–9.4	8.0	7.0–8.3
Supervisor report on clinical allocations (b)	10	8.1	7.5–8.2	8.3	7.5–9.1	8.0	7.6–8	8.0	7.7–8.3	8.5	7.0–8.7
Educational meetings (c)	20	17.5	17.3–17.5	16.0	15.8–20.0	17.0	8.0–17.2	19.0	14.0–20.0	20	0.0–20.0
Observations of the registrar (d)	10	10.0	10.0–10.0	9.1	8.0–10.0	10.0	8.3–10.0	9.3	8.1–10.0	8.0	7.0–8.7
Written assignments (e)	10	6.7	6.6–6.8	6.6	5.8–6.8	6.3	6.3–7.1	6.5	5.8–7.1	6.8	5.5–7.2
Logbook (f)	30	2.5	0.0–22.0	27.0	0.0–29.0	14.0	8.0–20.0	27.7	23.7–30.0	21.5	0.0–21.5
Global rating (g)	10	7.0	6.0–8.0	7.0	6.0–7.0	6.0	6.0–6.0	6.5	6.0–7.0	7.0	4.0–8.0
Learning Portfolio total scores (Total of items a-g)	100	62.1	58.8–75.6	81.0	53.9–88.1	64.0	63.8– 69.0	83.5	81.2–88.89	77	31.5–81.0
Quarterly assessment	100	67.1	66.0–73.2	67.5	55.7–72.9	62.6	60.0–63.8	65.4	62.2–68.3	62.0	54.8–70.8

IQR, interquartile range.

In the first- and second-year summative assessments (AoL), 6 out of 12 registrars passed the 18-month university examinations on the first attempt; five of the 12 failed them, and one registrar did not take the mid-point examinations. Of the six registrars who completed the 3 years of training, four who sat the national exit examination passed on their first attempt, and two did not take the examination.

## Clinical skills competence levels

The logbook skills section was incomplete across all training years, but primarily in the first year: four first-year registrars did not complete the section. The self-assessed competence levels of 18 registrars for 205 core clinical skills and 39 elective skills varied considerably (Appendix 1). Depending on the registrars’ self-assessment and the type of skill, competence levels of skills varied and scored from A to D (Appendix 1). Third-year registrars self-assessed their competence as higher (D scores) in most skills than in the other 2 years. The registrars reported higher competence levels in clinical domains such as adult health, women’s health, child health and emergency care than ENT, eyes and skin, orthopaedics and anaesthetics. First- and second-year registrars scored low in domains like orthopaedics, anaesthetics, ENT, eyes and skin because these clinical rotations occur during their third year of training. Most registrars, even first-year registrars, reported higher competence levels in consultation, clinical administration, clinical governance, COPC and teaching and learning domains (Appendix 1).

Although the self-reported level of competence in performing clinical skills was relatively better among third-year registrars compared to Years one and two, some core skills still included levels A, B and C. Examples included a laparotomy for ectopic pregnancy, cricothyroidotomy, assisted vaginal delivery, proctoscopy, vasectomy and reduction of elbow dislocation (Table 3). The total number of registrars in each year was six but varied as registrars did not report on many core skills and left them incomplete (Table 3).

**TABLE 3:** Self-reported competence levels on selected core clinical skills among third-year registrars (*n* = 6).

Skills	A: Only theoretical knowledge	B: Observed the skill	C: Performed under supervision	D: Fully entrusted to perform independently	Not reported
Cricothyroidotomy	0	1	3	1	2
Laparotomy for ectopic pregnancy	0	1	3	1	2
Assisted vaginal delivery	0	2	2	1	1
Elbow dislocation	0	1	2	2	1
Incision and drainage perianal abscess	0	0	3	2	1
Perform cardiac pacing	0	1	0	3	2
Apply club foot cast	0	2	1	1	2
Proctoscopy	1	0	3	1	1
Spirometry	0	2	1	2	1
Vasectomy	1	3	1	0	1
Culdocentesis	0	3	0	1	2
Brachial block	1	1	2	1	1

Table 4 shows the total skills set score medians and interquartile ranges for all 14 clinical domains in the LP across the 3 years of training. These medians were higher in adult health, women’s health, emergency care and child health for second- and third-year registrars than ENT, eyes and skin, orthopaedics and anaesthetics domains (Table 4). The Year two median was higher than Year three for the COPC and teaching and learning domains. When total core skills median scores progressed across the training years, elective skills median scores remained low across all three training years (Table 4).

**TABLE 4:** Total skills set score median and interquartile ranges for Years 1–3 (*N* = 18) in 14 family medicine domains.

Domain	1† (*n* = 6)		2 (*n* = 6)		3 (*n* = 6)	
	Median	IQR	Median	IQR	Median	IQR
Adult health (*n* = 38)	0	0–21	20.0	10–23	23.0	14–28
ENT, eye and skin (*n* = 22)	0	0–10	5.0	1–7	12.0	7–18
Women’s health (*n* = 30)	0	0–21	12.5	8–18	20.0	3–23
Consultation (*n* = 14)	0	0–14	10.5	6–12	13.0	8–14
Emergency care (*n* = 32)	0	0–28	27.0	19–32	28.0	16–29
Orthopaedics (*n* = 14)	0	0–7	1.0	0–9	8.0	2–9
Anaesthetics (*n* = 16)	0	0–8	0.5	0–5	13.0	12–14
Child health (*n* = 12)	0	0–2	10.0	9–10	11.0	10–12
Clinical administration (*n* = 6)	0	0–0	5.5	5–6	5.5	5–6
Forensics (*n* = 4)	0	0–3	2.0	2–4	3.5	3–4
Palliative care (*n* = 3)	0	0–0	0.0	0–0	1.0	0–2
Clinical governance (*n* = 8)	0	0–2	3.5	1–6	4.0	0–8
COPC (*n* = 3)	0	0–3	2.5	0–3	1.0	0–2
Teaching and learning (*n* = 9)	0	0–4	3.5	2–5	3.0	0–5
Core skills total (*N* = 205)	0	0–138	104.0	81–115	145.5	105–172
Elective skills (*N* = 39)	0	0–2	2.0	0–6	13.5	8–22

ENT, ear, nose and throat; COPC, community-oriented primary care; IQR, interquartile range.

† , 4/6 Year 1 registrars did not complete the logbook, scoring zero as lower quartile in all total skills set score medians. The total skills set score is the number of self-assessed D-scores in each domain.

## Discussion

This study examined the evidence of registrar learning from scores and self-reported clinical-skills competence levels recorded in LPs and QAs. The major finding was that LPs are not being used optimally as self-directed learning tools in decentralised training contexts, as has been found previously.^13,17,42^ The scores in various LP sections and skills level scores (for each skill and skill set) were variable in different clinical domains across training years and lacked adequate evidence of registrar progression in knowledge and skills. Although the LPs showed evidence of registrar learning, they can be used more effectively in WPBA.

Multiple assessments from various sources in authentic contexts improve assessment validity and reliability.^22^ Although there was evidence that the programme included multiple WPBAs, there was also evidence of lenient scoring in LP sections like the learning plans and skills competence levels. The negative impact of lenient scoring may be exacerbated by inadequate narrative feedback, as previously reported for this training programme.^35^ These combined problems raise concerns about whether LPs are being used effectively to support self-directed learning and suggest that supervisors need more clarity on using LPs in postgraduate training.^13,17^

The total LP scores improved across training years, but in QA scores across 3 years, second-year registrars had a higher score average than third-year registrars, similar to previous studies that detailed the inadequacies and leniency of supervisors while scoring WPBAs.^43^ Other LP sections, such as educational meetings and direct observations, showed the required number of educational meetings and WPBAs between supervisors and registrars. As stated in previous studies,^28,34,42^ registrars and supervisors in this study focused on completing LP sections rather than effectively utilising them as a learning tool. Whether supervisor–registrar engagement was quantified or engagement took place according to the expected standards with quality feedback to the registrars needs further exploration. In our WBL context, all LP scores were allocated by immediate FP supervisors and later verified by the university’s portfolio committee. The LP introduction in the first year, with adequate registrar training on its use and ongoing mentoring by supervisors, could have enhanced LP utilisation during self-directed learning.^21,44^

Quarterly assessment scores, part of the AfL component, contributed separately from LP scores to decide yearly registrar progression. In authentic clinical settings, supervisors and registrars struggle to use WPBA tools such as mini-CEX strictly as a summative tool during QA, always adding a formative component to those assessments. Similar challenges were identified related to mini-CEX use in high-income countries.^24,43^

The self-assessed competence in this study ranged across all four levels. Self-assessment is a feasible method for registrars to report their perceived competence, but it is susceptible to over- or under-reporting.^45,46^ Although the logbooks ensure that trainees perform the maximum number of skills required for competence,^23^ incomplete entries across all three training years undermine that intention. Registrars may have been reluctant to complete the logbook skills section because they perceived their skills competence as inadequate. Alternatively, they may not have felt confident about reporting their competence levels because of inadequate exposure to these skills in their context. Despite the increasing emphasis on self-assessment and critical analysis of trainees’ performance in competency-based medical education,^47^ the registrars appeared to lack the skills to self-assess their performance. The reported competence levels improved in higher training years, suggesting continuous skills learning at their workplaces. Registrars perform skill rotations in major disciplines during their first and second years of training, allowing them to acquire all needed skills. It was encouraging to find that registrars reported adequate competence levels in domain-specific skills learnt earlier in their training, such as consultation, clinical governance and administration, community-oriented primary care and teaching and learning.

A concerning finding was that specific skills such as cricothyroidotomy, assisted vaginal delivery, laparotomy for ruptured ectopic pregnancy and reducing an elbow dislocation were some examples of skills in which third-year registrars reported lower skills competence levels. Third-year registrars should be fully entrusted to perform these essential skills and be able to train junior registrars or students. Registrars’ skills deficiencies should be included in learning plans as learning needs, discussed with supervisors and planned on how to acquire them. Achieving adequate procedural skill competence levels by registrars is a priority in full-time postgraduate FM training programmes compared to older part-time programmes.^44^ The medical officers working in district hospitals in similar contexts reported adequate competence in performing these procedures.^45^ Once qualified as an FP, the registrar will need to function as a consultant and capacitator for medical practitioners and other health workers by performing various surgical and obstetric skills to strengthen district hospital services.^48^ This study reiterated that registrars require adequate exposure to learning opportunities to achieve mastery in performing core procedures encountered commonly in primary-care settings.

Third-year registrars reported lower competence levels on core clinical skills such as proctoscopy, applying clubfoot cast, vasectomy, culdocentesis and performing a brachial block. Questions that need to be answered include whether registrars had sufficient opportunities or allocated time to practise these skills during clinical rotations and whether these procedures were performed in sufficient numbers in various disciplines of the regional and district hospitals where registrars rotated. Current trends in medical education emphasise contextualising the curriculum and learning opportunities to acquire knowledge and skills needed in the health care system where trainees practice.^49^ While the FM skills list was revised in 2017,^36^ some core skills are still not performed sufficiently at peri-urban district hospitals.^45,50^ Perhaps it is time to revise the FM registrar training skills list to include more relevant context-specific procedural skills.

The impact of the training context on achieving the required levels of core skills competence may not be sufficiently considered. Family physicians in SA work in various health-system contexts like district, regional and tertiary hospitals, private general practice or rural and urban clinics, where they require most of these skills.^51^ Mastering all core skills could be more relevant for an FP practising in a rural district hospital where acute health worker shortages and referral challenges are experienced than in urban settings. It may be time to consider separating skill sets for FPs practising in rural and urban contexts.^45,52^ Even in high-income countries, doctors working in rural areas reported higher skill levels than their urban counterparts.^46,53^ A compulsory rural block for registrars working in peri-urban district hospitals to achieve specific skills, for example, a 2 to 3-month rotation or even a year of longitudinal clinical work from a peri-urban district hospital to a rural district hospital, could potentially mitigate the skills gaps identified among registrars.

Although studies were conducted when the LP was introduced into postgraduate FM training in SA about 10 years ago,^42^ there has not been further research to measure its longitudinal impact. This study initially sought evidence for WBL in the short term, but comparing the findings to studies from a decade ago highlights the long-term impact after LPs were introduced whether they were effectively utilised in WBL. Despite the positive impact of LPs on WBL, this study highlighted several issues that adversely affected how effectively registrars used the LPs, including incomplete logbook sections and a lack of clarity about the roles of FPs.

Workplace-based assessments captured in the LP provide trainees with more opportunities to reflect on their practice in authentic settings, positively influencing learning behaviour.^43^ Teamwork, professionalism and self-appraisal are all assessed when a mini-CEX tool is used in WPBAs.^22^ The assessment of complex tasks relied on the trainee’s ability to integrate cognitive, psychomotor and affective components, best evaluated in authentic clinical settings.^22^ Currently, the LP primarily focuses on scoring systems to determine whether the numbers needed are met or whether registrars achieved adequate skill competence levels. Providing more qualitative feedback on registrar performance will enhance the comprehensiveness of WPBAs. Most importantly, creating a learning environment that encourages a reflective dialogue between trainees and their supervisors is vital for effective LP utilisation.^20^

### Limitations

The number of LPs assessed was low, despite including all those available. However, the results contributed to a better understanding of the ‘phenomenon of interest’ for the broader mixed-methods case study, namely the postgraduate FM registrar decentralised training at the University of the Witwatersrand.

The data extracted depended on the legibility and completeness of various LP sections. Many skill competencies were incomplete, especially in the first-year registrar portfolios, with data collection likely impacted by the coronavirus disease 2019 (COVID-19) lockdown. During the lockdown, registrars struggled to complete LPs in some clinical skills rotations, which affected our results.

Self-assessment may have resulted in the over- or under-reporting of skills competency levels. However, this effect may have been mitigated by supervisors’ evaluating competency levels by directly observing procedures. Additional mitigation likely arose during the University’s portfolio committee’s final evaluation. All districts are represented on this internal committee, which interrogates the LPs and discusses inconsistencies, thereby improving the validity of the results. The initial intention was to determine the association of the scores and skills with the training districts and training years, but this was not feasible given the small number of LPs. Future research on entrusted decisions by supervisors on observed skills may corroborate registrars’ self-reported competency levels.

### Recommendations

The study highlighted the need for faculty development and registrar training to improve FPs’ and registrars’ literacy in WPBAs. Training will capacitate supervisors to mentor registrars effectively and enhance registrars’ self-directed learning. The LPs should include multiple assessments of various competencies, with both hard and soft skills, from various assessors in different contexts. Adequate formative feedback needs to be provided to augment registrar learning opportunities. Regular formative assessment visits by faculty programme managers will improve and maintain WPBA standards, which could translate to better registrar learning at their workplaces.

## Conclusion

This study aimed to evaluate evidence of learning in LPs, formative and summative WPBAs and the influence of the training district and the year of training on assessments. While the findings provided a holistic view of WPBA in FM training in one setting, they could apply to similar contexts at South African universities. Future research on WPBA across multiple training programmes will give a complete picture of postgraduate FM training in SA, which may also benefit other sub-Saharan African countries.

## Appendix 1

**TABLE 1-A1:** Self-reported skills competence levels on 205 core clinical skills in 18 registrar learning portfolios.

Domain	Skills (*N* = 18)	Category A A = only theoretical knowledge theory (*n*)	Category B B = Observed the skill (*n*)	Category C C = Performed under supervision (*n*)	Category D D = Fully entrusted to perform independently (*n*)	Not reported/left blank
**Adult health**						
*Perform side room tests*	Use a glucometer	0	0	0	11	7
	Use a haemoglobinometer	0	0	1	11	7
	Perform a pregnancy test	0	0	0	12	6
	Perform urinalysis	0	0	0	12	6
	Venepuncture	0	0	0	11	7
*Adult health general*	Femoral vein puncture	0	0	0	11	7
	Lumbar puncture	0	0	0	11	7
	Routine intravenous access	0	0	0	11	7
	Lymph node excision biopsy	1	0	3	7	7
	Perform point-of-care testing	0	0	1	11	7
*Adult musculoskeletal*	Measure shortening of the legs	0	0	2	9	7
	Aspirate and inject the knee joint	0	1	2	5	10
	Inject tennis elbow or golfer’s elbow	0	3	1	5	9
	Interpret radiographs of joints and bones	0	1	1	9	7
	Inject carpal tunnel syndrome	0	4	2	2	10
	Inject De Quervain’s tenosynovitis	0	4	1	3	10
	Inject the shoulder and subacromial bursa	1	2	2	3	10
	Inject trochanteric bursitis	1	4	1	2	10
*Adult abdomen*	Incision and drainage of perianal haematoma	0	1	4	5	8
	Interpret the abdominal radiograph	0	0	0	13	5
	Proctoscopy	1	2	4	2	9
	Interpret barium swallows	0	2	3	4	10
*Adult chest*	Electrocardiogram set up. record and interpret	0	0	0	13	5
	Interpret chest radiograph	0	0	0	13	5
	Measure peak expiratory flow	0	2	0	11	5
	Nebulise a patient	0	0	0	13	5
	Pleural tap	0	0	2	9	7
	Use inhalers and spacers	0	0	1	12	5
	Perform and interpret exercise stress test	0	5	0	4	9
	Perform and interpret office spirometry	0	4	2	3	9
*Adult urology*	Penile block	0	0	2	8	8
	Reduce a paraphimosis	0	1	1	9	7
	Male medical circumcision	0	3	3	5	8
	Drain hydrocele	0	3	2	4	9
	Insert a urinary catheter	0	0	0	11	7
	Insert a suprapubic catheter	0	0	3	9	6
	Interpret intravenous pyelogram	1	2	1	3	11
	Vasectomy	2	4	2	1	9
**ENT, eyes, skin**						
*Eyes*	Excision of chalazion	2	2	1	1	12
	Use a Schiotz tonometer	3	1	0	3	11
	Fundoscopy	0	1	1	7	9
	Instil drops or apply ointment	0	0	0	10	8
	Remove foreign body from the eye	0	1	2	5	10
	Test for squint	0	1	2	4	11
	Washout of eyes	0	0	1	7	10
*ENT*	Assess hearing loss	0	0	2	7	9
	Reduce a fractured nose	2	2	1	3	10
	Remove a foreign body from ear and nose	0	0	1	10	11
	Syringe. dry swab an ear	0	0	0	10	8
	Take a throat swab	0	0	0	11	7
	Manage epistaxis	0	0	1	1	16
	Suture a pinna lobe	0	0	1	9	8
	Drain a peritonsillar abscess	0	4	2	3	9
*Skin*	Inject keloids	0	1	3	3	12
	Phenol ablation of ingrown toenail	2	1	2	1	12
	Excise sebaceous cyst	0	1	2	2	12
	Compression dressing to venous leg ulcer	0	0	3	7	8
	Cryotherapy or cauterisation	1	1	0	5	12
	Skin biopsy	0	1	3	3	12
	Wide-needle aspiration biopsy lymph node	1	0	1	6	10
**Women’s health**						
*Pregnancy*	Obstetric ultrasound	0	0	1	9	8
	Interpret antenatal growth chart	0	0	0	11	7
	Assess foetal well-being during labour	0	0	0	11	7
	Episiotomy and suturing	0	0	2	9	7
	Examine progress during labour and use partogram	0	0	1	10	7
	Normal vaginal delivery	0	0	1	10	7
	Apply and interpret the cardiotocograph	0	0	2	9	7
	Assess foetal movement and counsel use of kick chart	0	0	0	10	8
	Assisted vaginal delivery	0	3	2	5	8
	Caesarean section and management of bleeding	0	1	3	8	6
	Evacuation of uterus	0	0	0	9	9
	Manual removal of placenta	0	0	1	9	8
	Repair of third-degree tear	0	2	3	5	8
	Pelvic ultrasound	0	0	2	9	7
*Woman’s health*	Culdocentesis	1	4	0	2	11
	Hormone implants	0	2	3	7	6
	Laparotomy for ectopic pregnancy	0	3	4	3	8
	Termination of pregnancy	0	2	2	7	7
	Insertion of intrauterine contraceptive device	0	2	3	7	6
	Papanicolaou smears	0	0	0	14	4
	Dilatation and curettage	0	0	1	9	8
	Drainage Bartholin’s abscess or cyst	1	0	2	9	6
	Endometrial biopsy	0	2	1	8	8
	Fine-needle aspiration biopsy of breast lump	1	1	1	7	8
	Tubal ligation	0	0	2	8	8
	Cervical polyp removal	0	3	3	2	10
*Newborn*	Assess gestational age at birth	0	0	0	12	6
	Counsel on Kangaroo mother care	0	0	0	11	7
	Resuscitate a newborn	0	1	0	12	5
	Umbilical vein catheterisation	0	0	0	13	5
*Consultation*	Patient-centred consultation	0	0	1	13	4
	Use genogram and eco-map	0	0	0	14	4
	Develop and use flowcharts for chronic care	0	2	1	8	7
	Motivate behaviour change	0	0	2	12	4
	Assess and consult families. couples	0	0	1	12	5
	Shared consultation to capacitate nurse practitioner	0	0	1	13	4
	Counselling skills for HIV. Termination of pregnancy, sexual assault	0	0	2	12	4
	Break bad news	0	0	1	12	5
	Mini–Mental State Examination	0	0	1	10	7
	Use problem-orientated medical record	0	0	1	10	7
	Conduct a family conference	0	1	3	8	6
	Cope with language barriers	0	0	2	11	5
	Holistic assessment and management	0	0	1	12	5
	Sexual history and counselling	0	0	2	9	7
**Emergency care**						
	Calculate % burn	0	1	12	0	5
	Manage choking	0	0	3	7	8
	Prescribe oxygen using a variety of devices	0	0	0	13	5
	Immobilise the spine	0	0	0	11	7
	Insert an advanced airway Endotracheal tube, laryngeal tube	0	0	0	13	5
	Measure the Glasgow Coma Scale	0	0	1	12	7
	Administer rabies prophylaxis	0	0	0	12	7
	Advanced cardiopulmonary resuscitation – Adult	0	0	0	13	5
	Advanced cardiopulmonary resuscitation– Child	0	0	1	8	9
	Debride wounds or burns	0	0	1	12	5
	Gastric lavage	0	0	1	9	8
	Give a blood transfusion	0	0	0	13	5
	Incision and drainage of abscesses	0	1	0	12	5
	Insert chest drain	0	0	0	13	5
	Insert nasogastric tube	0	0	0	13	5
	Interpret radiographs in trauma	0	0	1	11	6
	Emergency venous access	1	0	2	9	7
	Manage snake bite	1	2	2	6	8
	Primary survey	0	0	0	13	5
	Relieve tension pneumothorax	0	0	1	10	7
	Remove a foreign body from skin	0	0	2	8	8
	Secondary survey	0	0	0	13	5
	Selecting emergency equipment for doctor’s bag	0	0	1	12	5
	Debride and suture lacerations	0	0	0	13	5
	Prepare and stabilise a critically ill patient for transport	0	0	2	11	5
	Cricothyroidotomy	1	2	3	4	8
	Insert central line	0	1	2	6	10
	Connect a patient to a ventilator and monitor the patient	0	2	0	10	6
	Perform cardiac pacing	0	2	2	7	7
	Perform synchronised cardioversion	0	1	1	9	7
	Perform arterial sampling: adult and child	0	0	1	11	8
	Classify patient according to triage system	0	0	0	10	8
**Orthopaedics**						
	Apply finger and hand splints	0	1	2	8	7
	Apply casts to upper and lower limb	0	0	2	8	8
	Closed reductions on hand. forearm. tibia. fibula	0	1	2	8	7
	Set up skeletal and skin traction	1	0	1	8	8
	Reduce elbow dislocation	0	1	3	6	8
	Reduce hip dislocation	0	2	3	5	8
	Reduce radial head dislocation	0	2	2	5	9
	Reduce shoulder dislocation	0	0	0	11	7
	Excise ganglion	1	0	3	5	9
	Amputations – fingers	1	1	2	3	12
	Apply club foot cast	1	3	1	2	11
	Debridement of open fractures	0	1	1	6	10
	Emergency fasciotomy	1	2	1	1	12
**Anaesthetics**						
	Injections – intra-dermal. subcutaneous. Intramuscular	1	0	0	8	11
	Ring block	1	0	0	10	7
	Check Boyle’s machine	1	0	0	7	10
	Control airways with mask	1	0	0	10	8
	General anaesthetic	1	0	0	6	11
	Intubate and ventilate patient	0	0	0	11	7
	Ketamine anaesthesia	1	0	0	8	9
	Monitor patient during anaesthetic	1	0	1	6	9
	Monitor patient during recovery	1	0	0	6	11
	Reverse muscle relaxation	1	0	1	5	11
	Select an appropriate circuit – Magill Circle. T-piece	1	1	0	6	10
	Spinal anaesthetic	1	0	0	6	11
	Ventilate patient using mask and bag	0	0	1	6	11
	Bier’s block	1	3	2	1	11
	Brachial block	2	1	3	1	11
	Administer conscious sedation and monitor	0	1	0	8	9
**Child health**						
	Assess growth and classify malnutrition	0	0	0	11	7
	Capillary blood sampling	0	0	0	10	8
	Assess chest radiograph in child	0	0	0	12	6
	Developmental assessment	0	0	1	10	7
	How to do, interpret Tine and Mantoux tests	0	0	0	12	6
	Intraosseous line	0	0	1	9	8
	Intravenous access in a child	0	0	0	12	6
	Lumbar puncture in a child	0	0	1	11	6
	Manage problems using the integrated management of childhood	0	0	0	11	7
	Suprapubic bladder puncture	0	1	3	5	9
	Venepuncture – upper limb and external jugular vein	0	0	1	9	8
	Manage neonatal jaundice with phototherapy	0	0	2	8	8
**Clinical administration**						
	Complete sick certificates	0	0	0	12	6
	Complete death certificates	0	0	0	12	6
	Certify patient under *Mental Health Care Act*	0	0	1	8	9
	Writing appropriate referral letters	0	0	0	12	6
	Managing a clinic for chronic care	0	0	0	12	6
	Perform work assessment and complete disability grant	0	0	1	11	6
**Forensics**						
	Assess. manage and document drunken driving	0	0	0	12	6
	Assess. manage and document interpersonal violence	0	0	2	10	6
	Assess, manage and document sexual assault	0	0	2	7	9
	Complete J-88 form following assault	0	0	0	13	5
**Palliative care**						
	Counselling of a dying patient	0	0	3	4	11
	Hypodermoclysis (subcutaneous infusion)	2	1	2	2	11
	Set up a syringe driver	0	0	2	5	11
**Clinical governance**						
	Contribute to the development or revision of guidelines	0	0	3	5	10
	Facilitate the implementation of clinical guidelines	0	0	1	8	9
	Improve quality of care by facilitating QIP	0	0	4	8	6
	Improve cost-effectiveness by rational prescribing	0	0	1	8	9
	Build capability and quality care by teaching, training, and mentoring	0	0	3	9	6
	Critically appraise new evidence	0	0	1	8	9
	Appraise the competence of new clinicians and set appropriate levels of independence versus support	0	0	2	6	10
	Evaluate the quality of care in relation to the relevant clinically orientated national core standards	0	0	4	4	10
**COPC**						
	Do a home visit	0	0	2	8	8
	Make a community diagnosis	0	0	2	8	8
	Promote health in communities	0	0	1	8	9
**Teaching and training**						
	Plan and implement a teaching or continuing professional development activity	0	0	1	9	8
	Use a portfolio of learning	0	0	1	8	9
	Mentor a colleague	0	0	2	7	9
	Facilitate small group learning	0	0	1	10	7
	Prepare and give a presentation	0	0	1	11	6

Note: Number of registrars that achieved maximum in each category (A–D), Incomplete = no answer recorded or left blank.
